# A Late-Onset Presentation of Miyoshi Myopathy: A Case Report

**DOI:** 10.7759/cureus.87683

**Published:** 2025-07-10

**Authors:** Ubaid Ansari, Aakriti Dua, Esha Aneja, Noorhan Amani, Zohaer Muttalib, Forshing Lui, Biljinder S Chima

**Affiliations:** 1 Family Medicine, California Northstate University College of Medicine, Elk Grove, USA; 2 Pediatrics, California Northstate University College of Medicine, Elk Grove, USA; 3 Psychiatry, California Northstate University College of Medicine, Elk Grove, USA; 4 Internal Medicine, California Northstate University College of Medicine, Elk Grove, USA; 5 Clinical Sciences, California Northstate University College of Medicine, Elk Grove, USA; 6 Family Medicine, Sports Medicine, Rocklin Family Practice and Sports Medicine, Rocklin, USA

**Keywords:** creatine kinase, dysferlin, dysferlinopathy, miyoshi myopathy, muscular dystrophy

## Abstract

Miyoshi myopathy is a muscular dystrophy disease characterized by muscle weakness and atrophy generally in distal muscle groups, such as in the legs and arms. Miyoshi myopathy is thought to occur due to genetic mutations in the DYSF gene, which codes for the dysferlin protein, which is critical for muscle cell membrane integrity and muscle fiber adhesiveness. The first symptoms begin in early adulthood and include weakness and atrophy in the calves, gait abnormalities, pain and discomfort in affected muscles, and difficulty jumping or walking on tiptoes. Patients generally are diagnosed by a combination of physical exam findings, genetic testing, muscle biopsy, and elevated creatinine kinase (CK) levels. Management of the disease progression includes physical therapy to strengthen the muscles, nutritional support, occupational therapy, and assisted device education. While not life-threatening, Miyoshi myopathy outlook is generally considered moderate to poor due to significant muscle weakness and eventual loss of mobility usually in 10-20 years after onset. We present a unique case of a 66-year-old male patient complaining of pain in his bilateral calves after having had a series of back surgeries 10 years prior. A diagnosis of Miyoshi myopathy, a rare occurrence in this age group, was made based on CK levels. In this report, we will discuss the pathophysiology, disease progression, and management of Miyoshi myopathy.

## Introduction

Miyoshi myopathy is a rare autosomal recessive muscular dystrophy that primarily affects distal muscles. It was first described in 1967 by Miyoshi et al., who identified four patients from two different families in Japan presenting with a distal myopathy that appeared to follow an autosomal recessive inheritance pattern [1]. Subsequent cases were reported in Finland, South Korea, Brazil, and Japan [2]. The observed changes were similar to those in Duchenne muscular dystrophy, with muscle biopsies showing segmental necrosis, myopathic changes, and signs of regeneration. Miyoshi et al. classified Miyoshi myopathy within the family of dysferlinopathies, caused by mutations in the DYSF gene, which encodes the dysferlin protein. Dysferlin is essential for repairing skeletal muscle cell membranes after damage [1].

The most prominent symptoms of Miyoshi myopathy include muscle weakness and atrophy, primarily affecting the distal limbs [3]. The gastrocnemius and soleus muscles are particularly impacted, though muscle weakness can progress to involve the gluteal and thigh muscles [4]. Characteristic changes include difficulty standing on toes, with minimal impairment in standing on heels [3]. Progressive muscle weakening can lead to wheelchair dependence in some cases, and patients may experience challenges climbing stairs or fatigue with walking [4]. Additional difficulties include ankle dorsiflexion and forearm atrophy, which can reduce grip strength [3].

Miyoshi myopathy typically presents in young adulthood, between 15 and 30 years of age, with a broader range of onset from 15 to 36 years [2]. It is inherited in an autosomal recessive pattern and is caused by mutations in the DYSF gene located on chromosome 2p13. This mutation disrupts dysferlin function, leading to increased muscle cell damage [5]. Diagnosis is confirmed using genetic testing, immunohistochemistry, and electromyography. A significant increase in serum creatine kinase (CK) levels, up to 25-150 times the normal range, is often observed [2]. Elevated CK levels may also be detected in preclinical stages and in some heterozygous carriers of the gene mutation [3]. Early involvement of the gastrocnemius muscles, accompanied by pain and discomfort in the calves, is a key clinical hallmark for diagnosis [2]. Muscle MRI can also aid in diagnosis and tracking disease progression, revealing characteristic patterns of muscle involvement [6].

This case highlights an atypical presentation of Miyoshi myopathy in a 66-year-old male patient, emphasizing the importance of considering Miyoshi myopathy in patients with isolated calf atrophy and elevated CK levels, even in the absence of typical age at onset. The diagnostic challenges and clinical course of this late-onset presentation are also discussed, which provides new insights into the variable phenotypic spectrum of this rare myopathy.

## Case presentation

A 66-year-old male patient presents with a chief complaint of progressive bilateral calf weakness and atrophy, which began approximately 10 years ago following a series of back surgeries. These surgeries included lumbar laminectomies in 2019 and 2023 for spinal stenosis, as well as a lumbar fusion at L4-L5 in 2014 due to persistent lower back pain. Radiography in 2024 indicated multilevel degenerative disc disease throughout the patient’s spine. Initially, the calf weakness was subtle, but over the past two to three years, the patient has noticed worsening calf atrophy and increasing difficulty with balance, leading to a noticeable limp. The weakness was noted to be worse on his right side but noted that his left side had also begun to progressively weaken. Despite seeing several surgeons who conducted various tests, there was no recovery of calf muscle function and he was informed that the muscles had not "woken up." There have been no sensory changes throughout the course of his symptoms.

**Figure 1:**
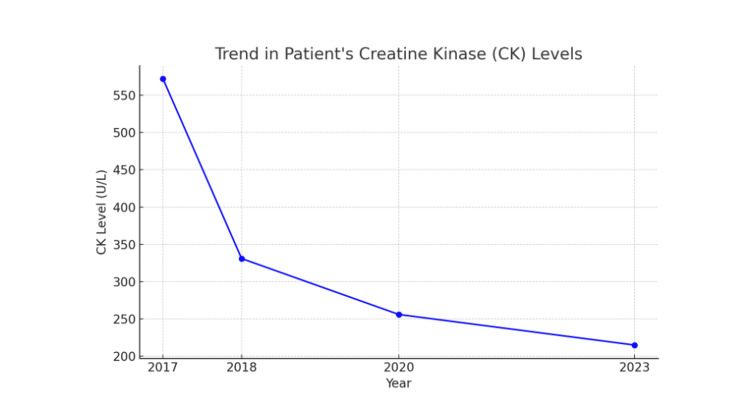
Trend in Patient’s Creatine Kinase (CK) Levels

The patient’s past medical history includes hypercholesterolemia, for which he was treated with statin therapy. During statin therapy, the patient’s CK levels were elevated, reaching a peak of 572 U/L in late 2017. Following discontinuation of statin therapy in 2018, his CK levels gradually declined, dropping to 331 U/L within the same year, then to 256 U/L in 2020, and eventually to 215 U/L by 2023 (Figure 1). However, his persistent calf atrophy and weakness did not resolve despite the normalization of CK levels. This suggested that statin-induced myopathy was unlikely to be the cause of his symptoms, pointing to an alternative etiology such as a structural myopathy. If statin-induced myopathy was the primary cause of his symptoms, improvement in muscle function or reversal of atrophy would typically be expected after discontinuation of the drug. Additionally, there is no known family history of neurological disorders, and other muscle groups remain unaffected.

**Figure 2:**
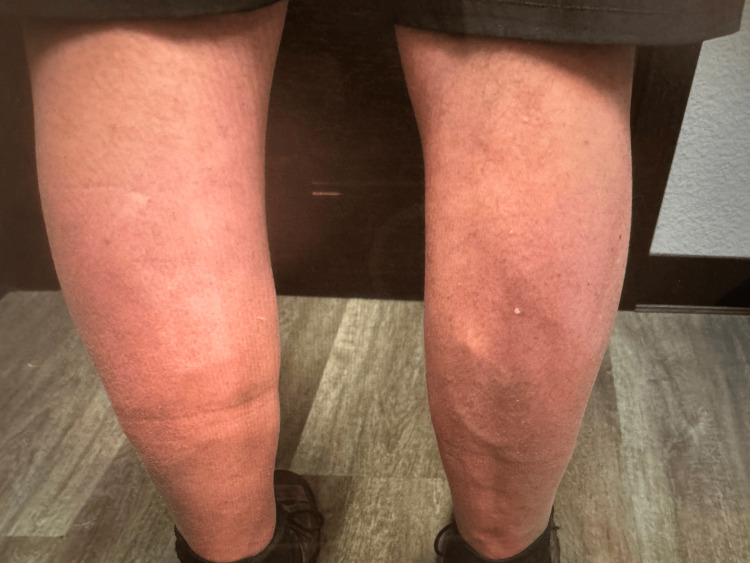
Bilateral Muscle Wasting in the Calves

Upon examination, the patient exhibits isolated bilateral calf atrophy without signs of sciatica or sensory loss (Figure 2). Given the lack of response to previous back surgeries, the absence of sensory involvement, and a history of elevated CK levels, a myopathic etiology was considered more likely than a neurogenic cause. Some tests were performed to figure out the etiology of his calf atrophy. Needle electromyography (EMG) demonstrated consistent findings of a myopathic process across multiple lower extremity muscles, particularly within the distal muscles. Short-duration, low-amplitude motor unit action potentials (MUAPs) and early recruitment patterns were noted in the gastrocnemius, peroneus longus, and tibialis anterior muscles (Table 1). These findings align with the characteristic involvement of distal muscles seen in Miyoshi myopathy. Additionally, spontaneous activity such as fibrillations was noted in the medial gastrocnemius bilaterally and right extensor hallucis longus, indicating ongoing muscle denervation or muscle fiber degeneration (Table 1). Electrodiagnostic studies showed bilateral L4 to S1 polyradiculopathy, most severely affecting the bilateral S1 myotome, with signs of ongoing denervation in bilateral gastrocnemius muscles. Nerve conduction studies (NCS) revealed preserved sensory nerve function bilaterally, with normal conduction velocities and amplitudes in both superficial peroneal and sural nerves (Table 2). This absence of sensory involvement is an important distinguishing feature, helping to rule out polyneuropathies or radiculopathies as the primary etiology. Genetic testing identified a homozygous mutation in the DYSF gene, confirming the diagnosis of Miyoshi myopathy. The neurologist diagnosed the patient with Miyoshi myopathy, a form of muscular dystrophy primarily affecting the calf muscles. It was emphasized that the atrophy is isolated to the calf muscles and is not expected to progress to other muscle groups (Figure 2).

**Table 1:** Needle Electromyography (EMG) Examination MUAPs: Motor unit action potentials; Gr. Incr.: greatly increased.

EMG	Insertional	Spontaneous Activity				Volitional MAUPs			
Muscle	Insertional	Fibrillations	Positive Wave	Fasciculations	Duration	Amplitude	Polyphasic	Configuration	Recruitment
Gastrocnemius Medial Head R	Normal	2+	None	None	Gr. Incr.	Gr. Incr.	None	Normal	Reduced
Peroneus Longus R	Normal	None	None	None	Gr. Incr.	Gr. Incr.	None	Normal	Reduced
Tibialis Anterior R	Normal	None	None	None	Gr. Incr.	Gr. Incr.	None	Normal	Reduced
Extensor Hallicus Longus R	Normal	2+	None	None	Gr. Incr.	Gr. Incr.	None	Normal	Reduced
Vastus Lateralis R	Normal	None	None	None	Normal	Normal	None	Normal	Normal
Hamstrings R	Normal	None	None	None	Gr. Incr.	Gr. Incr.	None	Normal	Reduced
Tibialis Anterior L	Normal	None	None	None	Gr. Incr.	Gr. Incr.	None	Normal	Reduced
Gastrocnemius Medial Head L	Normal	2+	None	None	Gr. Incr.	Gr. Incr.	None	Normal	Reduced

**Table 2:** Sensory Nerve Conduction Studies (NCS)

Sensory Nerve and Site	Onset Latency (ms)	Peak Latency (ms)	Amplitude (μV)	Segment	Latency Difference (ms)	Distance (mm)	Conduction Velocity (m/s)
Superficial Peroneal R (Ankle)	2.2	3	4	Dorsum of Foot-Ankle	2.2	100	45
Sural R (Lower Leg)	2.1	2.7	10	Ankle-Lower Leg	2.1	90	44
Superficial Peroneal L (Ankle)	2.3	2.8	3	Dorsum of Foot-Ankle	2.3	100	43
Sural L (Lower Leg)	2	2.6	10	Ankle-Lower Leg	2	90	45

At a follow-up visit with the family medicine physician, the patient remained ambulatory and reported no significant pain, except for mild discomfort in the wrist and first carpometacarpal (CMC) joint, attributed to arthritis. He was advised that no further testing was necessary, except for monitoring CK levels to assess any future changes.

## Discussion

This study reports the case of a patient with late-onset Miyoshi myopathy with symptoms beginning at the age of 51 years with no reported family history of distal muscle dystrophy. The patient’s elevated CK levels, inability to stand on tiptoes, and noticeable calf atrophy, with EMG studies depicting a lower muscle etiology and normal NCS studies, support the diagnosis of Miyoshi myopathy.

The typical onset of Miyoshi myopathy is between 15 and 30 years with patients experiencing initial symptoms of lower limb muscle weakness and difficulty climbing stairs or jumping at the age of 19 years [7]. However, our patient differs from the typical presentation and began experiencing calf weakness at the age of 48. Additionally, muscle biopsy indicated muscle fiber degeneration of the gastrocnemius with necrosis, which is characteristic in myopathies, though additional features can include fatty replacement or muscle cells, substantial mononuclear cell infiltration with dystrophic changes, and disrupted sarcolemma with thickened basal lamina caused by defects in dysferlin protein that maintains muscle membrane stability [8]. Muscle biopsy for this patient did not show any inflammatory infiltrates but immunohistochemistry showed an absence of dysferlin from muscle sarcolemma.

Interestingly, genetic testing did not show a homozygous mutation in the DYSF gene. Single-nucleotide variants of the DYSF gene were not reported for this patient, but compound heterozygous mutations in c.663+1G>C and p.Trp992Arg have been associated with dysferlinopathy [9]. In addition to mutations in the DYSF gene, which cause Miyoshi myopathy, mutations in anoctamin-5 (ANO5) have been noted to cause a similar presentation called Miyoshi myopathy type 3. ANO5, located on chromosome 11 at 11p14.3, is a transmembrane calcium-activated chloride channel that is expressed in various cells, including skeletal and cardiac muscle cells, chondrocytes, and osteoblasts [10]. Phenotypically, those with ANO5 mutations present with late-onset dystrophy with a mean age of onset between 32 and 41 years but can present even later in life with some cases presenting 70 years or older [11,12]. Patients with ANO5 will slowly develop asymmetric weakness and muscle atrophy, asymptomatic hyperCKemia, and exercise-induced myalgia [13]. While ANO5 mutations were not studied in this patient, the patient’s late onset of Miyoshi myopathy, asymmetric distal lower extremity weakness beginning on his right side, along with asymptomatic hyperCKemia could be attributed to an ANO5 mutation, but immunohistochemical studies may suggest otherwise. Immunohistochemical studies on muscle cells of patients with ANO5 mutations usually show a reduction or normal expression of dysferlin expression rather than a complete absence of the protein as was the case in our patient [14-16]. These can suggest that an ANO5 mutation is not the primary cause of our patient’s presentation, but that a mutation or other etiology affecting dysferlin expression is.

Before experiencing symptoms of Miyoshi myopathy, the patient had complained of back pain, ultimately receiving a lumbar laminectomy. Radiography and computed tomography (CT) imaging of the patient indicated disc space narrowing, spurring, and multilevel spondylosis. He was eventually noted to have multilevel degenerative disc disease, which contributes to the patient’s back pain. Another case reported a correlation between Miyoshi myopathy and rigid spine, showing early spinal contractures in a 23-year-old patient, which began with a weakness of gastrocnemius muscles at the age of 13. This patient had a c.4282 C>T mutation in the DYSF gene and immunohistochemistry, and Western blot analysis indicated no expression of dysferlin [17]. Our patient first presented with back pain and later with Miyoshi myopathy. While the two conditions may not be related, it is important to note that with the patient’s late onset, back pain can be an early indication for the disease.

Currently, there is no cure for Miyoshi myopathy, and treatment focuses on supportive care. Physical therapy plays a central role in improving muscle function and alleviating symptoms. Management strategies such as emotional support, weight management to prevent obesity, and stretching exercises to maintain muscle flexibility have been reported as beneficial. Steroids are the mainstay treatment for muscular dystrophies, potentially delaying disease progression. Patients with Miyoshi myopathy may remain ambulatory for 10-15 years before requiring assistive devices and typically need a wheelchair after 20-25 years [18].

Emerging therapies are exploring genetic and cell-based molecular approaches to repair muscle membranes. For example, a study by Zhou et al. demonstrated the use of adeno-associated virus vectors for delivering the DYSF gene, which successfully restored dysferlin function and improved skeletal muscle performance [19] Additionally, myogenic stem cell transplantation therapy has shown promise as a potential treatment, offering fewer adverse effects compared to exon skipping, stop codon read-through, and viral vector-based methods [20]. However, challenges such as the efficiency of engrafted cells and systemic delivery remain obstacles to widespread use.

Despite advancements in genetics, understanding of the molecular mechanisms underlying Miyoshi myopathy and the development of curative treatments remains limited. Additional research is necessary to elucidate genotype-phenotype correlations and to develop targeted therapies for the disorder. Given the rarity of Miyoshi myopathy, expanding the existing literature is essential to fully understand its pathogenesis and improve treatment options for affected patients.

## Conclusions

Miyoshi myopathy is a rare muscular dystrophy that has an autosomal recessive mode of inheritance and typically affects the muscles of the posterior compartment of the calf. Classically, Miyoshi myopathy presents earlier in life, and genetic testing of patients with the condition demonstrates a mutation in the DYSF gene. However, this particular case is unusual, as this patient did not have symptoms and was not diagnosed with Miyoshi myopathy until his 50s, and genetic testing did not reveal a homozygous mutation in the DYSF gene. This may suggest that Miyoshi myopathy may not always present in the same manner as commonly described in the literature; its onset could be later on in life, and it may not always be genetically inherited and could be due to other previously unexplored etiologies. Thus, it is imperative that Miyoshi myopathy is not ruled out as a differential diagnosis for a patient presenting with calf muscle weakness or atrophy, even if the patient does not fit into the expected age range or genetic profile, to ensure that patients are not underdiagnosed or misdiagnosed. 
